# Characterization of a novel *HESX1* mutation in a pediatric case of septo‐optic dysplasia

**DOI:** 10.1002/ccr3.868

**Published:** 2017-03-02

**Authors:** Sara Pozzi, Wen‐Hann Tan, JuanPedro Martinez‐Barbera

**Affiliations:** ^1^ Developmental Biology and Cancer Research ProgrammeBirth Defects Research CentreUCL Great Ormond Street Institute of Child HealthLondonUK; ^2^ Division of Genetics and GenomicsBoston Children's HospitalBostonMassachusettsUSA

**Keywords:** HESX1, hypopituitarism, septo‐optic dysplasia

## Abstract

Septo‐optic dysplasia (SOD) is a rare condition for which the precise etiology is still unclear. Elucidating the genetic component of SOD is a difficult but necessary task for the future. We describe herein a novel *HESX1* c.475C>T (p.R159W) mutation and demonstrate its potential pathogenicity in the development of this rare disease.

## Introduction

Septo‐optic dysplasia (SOD) is a congenital developmental anomaly of the brain, possibly a monotopic field defect, resulting in the combination of the absence of septum pellucidum (the “septo” component), optic nerve hypoplasia (the “optic” component), and hypopituitarism 1, 2. It affects one in 10,000 livebirths with a 1:1 ratio between females and males 3. This condition was first reported in 1941 by Reeves who described a patient with a combination of absence of the septum pellucidum and hypoplasia of the optic nerve 4. In subsequent years, more cases with the same defects were described, and in 1956, De Morsier termed this condition “septo‐optic dysplasia” 5. The pituitary component of this condition was later identified in 1970, when Hoyt et al. described clinical evidence of hypopituitarism in four children with radiological evidence of SOD 6. The spectrum of clinical manifestations in SOD is quite broad, but the three classical features are (1) abnormalities of midline structures (e.g., corpus callosum, septum pellucidum, and anterior commissure), (2) congenital hypothalamic‐pituitary insufficiency, and (3) optic nerve hypoplasia 1.

Septo‐optic dysplasia is considered a highly heterogeneous condition in which the precise etiology is still unclear and believed to be multifactorial with both environmental and genetic components 7. To date, a number of mutations in critical developmental genes that control brain morphogenesis have been identified as possible genetic causes of SOD 8. This includes the homeobox‐containing transcriptional repressor HESX1*,* an essential regulator of forebrain and pituitary development in mice 9, 10, 11 and humans 12, 13.

*HESX1* maps to chromosome 3p14.3 (human genome build: GRCh38), and its open reading frame (ORF) is highly conserved among different vertebrates 14. HESX1 proteins contain a homeodomain that is responsible for DNA binding, and two repressor domains, one in the N‐terminal (*eh1* and HRPW motifs) and one in the C‐terminal homeodomain 15. Although direct transcriptional targets for HESX1 remain unknown, HESX1 binds to an artificial consensus sequence (5′‐TAATYNRATTA‐3′) called P3 16, and repression is facilitated by the interaction of HESX1 with several corepressors (e.g., TLE/groucho) 15, 17, 18. Mice carrying *Hesx1* null or specific point mutations identified in SOD patients show forebrain and pituitary phenotypes that closely resemble human SOD, suggesting a conserved function for HESX1 in mammals 9, 19. Here, we report the identification of a novel *HESX1* p.R159W mutation in a case of SOD, which broadens our understanding of the genetic causes of this important and complex human condition.

## Subjects and Methods

### Study subject

**Figure 1:**
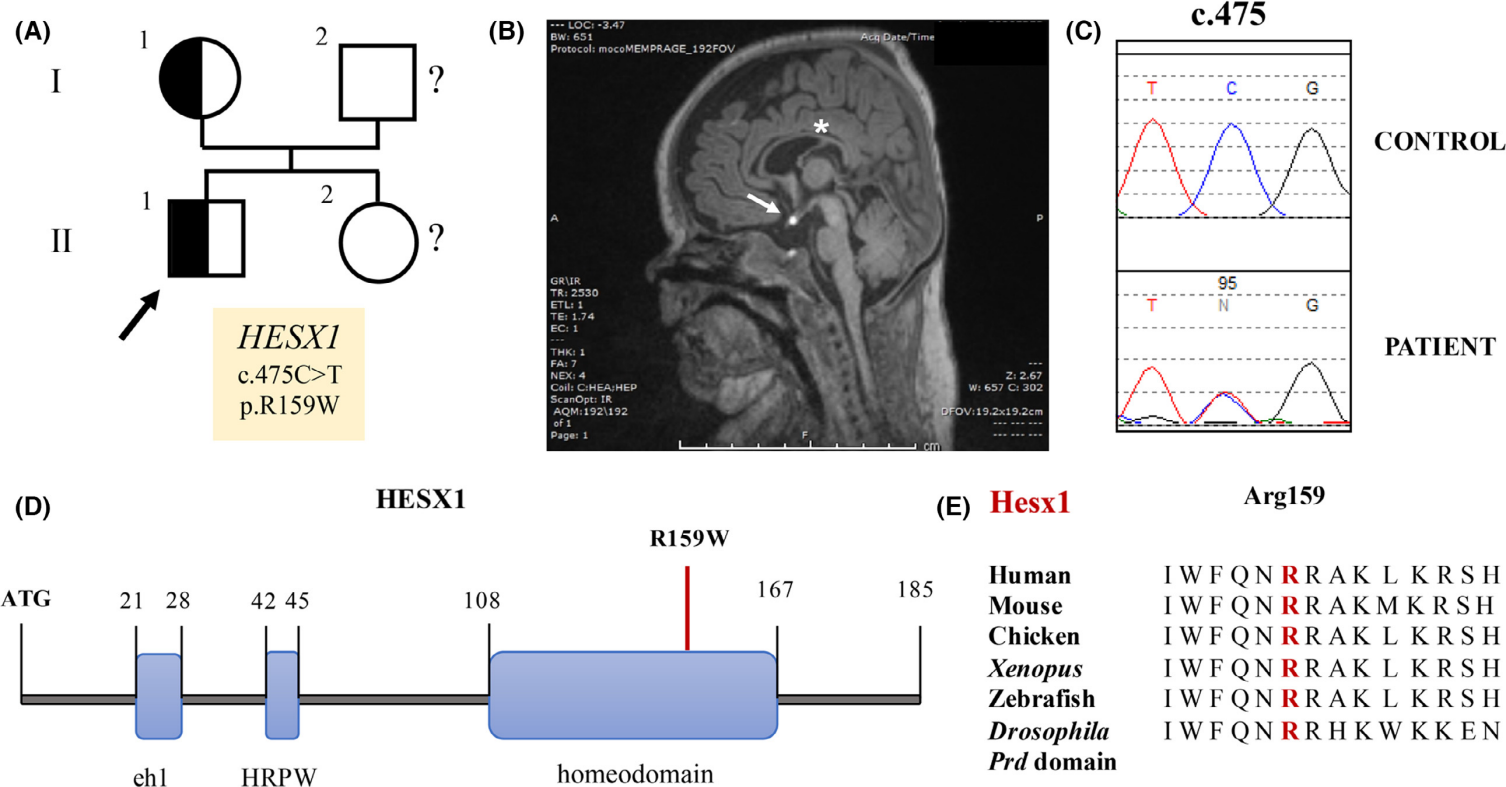
Identification of a *HESX1* c.475C>T (p.R159W) mutation in a patient with septo‐optic dysplasia. (A) The proband (arrow) and his mother were found to be heterozygous for c.475C>T. The father and the female sibling could not be screened. (B) MRI sagittal view of the proband brain. Note the thin corpus callosum (*) and the ectopic posterior pituitary (arrow), indicative of a failure to descend. (C) Electropherogram showing the heterozygous variant. Note the presence of the C>T mutation in the patient compared to a healthy control individual. (D) Schematic representation of HESX1 functional domains. The *eh1* and the HPRW domains at the N‐terminus are responsible for the interaction of HESX1 with corepressors of the TLE/Groucho family. The homeodomain at the C‐terminus mediates DNA binding and interaction with nuclear corepressor. The position of the p.R159W substitution is indicated. (E) Protein homology analysis of the third helix of the homeodomain region containing the R159W substitution. R159 has been conserved during evolution from flies to humans.

The proband, a male infant (II‐1; Fig. 1A), was born at 37 weeks’ gestation with a birth weight of 2.126 kg to a healthy 27‐year‐old mother. At 3 months of age, his length was 46.4 cm and his head circumference 34 cm, with a body weight of 3.74 kg. He had been diagnosed prenatally with gastroschisis and underwent multiple surgeries in the first 2 months of life to repair the gastroschisis and to manage the ensuing complications, including abdominal compartment syndrome and wound dehiscence. During that period and beyond, he also suffered from multiple urinary tract, respiratory tract, and central line infections; septic shock, conjugated and unconjugated hyperbilirubinemia, anemia, and thrombocytopenia. At about 4 months of life, the glucose infusion rate (GIR) in his total parenteral nutrition was about 14–15 mg/kg/min, but as his GIR was being weaned, he developed hypoglycemia with his blood glucose dropping to about 1.3 mmol/L. He had a normal response to the low‐dose ACTH (cosyntropin) stimulation test and did not have any biochemical evidence of adrenocortical insufficiency, although he responded well to short courses of stress‐dose corticosteroids intermittently. He was also noted to have growth hormone deficiency, for which replacement therapy or stimulation test was not considered given the acute illness of the proband, who never managed to leave the intensive care unit. The proband showed low free thyroxine (T4) and triiodothyronine (T3) levels, but normal thyroid‐stimulating hormone (TSH). There is no clinical evidence of hypogonadism. Moreover, he was noted to have circular pendular nystagmus. Electroencephalography (EEG) showed diffuse slowing suggestive of encephalopathy, but there were no epileptiform discharges nor was there any correlation with the abnormal eye movements.

Ophthalmologic evaluation at almost 5 months of age revealed bilateral optic nerve hypoplasia. Brain MRI performed shortly thereafter confirmed that the optic nerves and chiasm were hypoplastic; in addition, the left olfactory nerves, bulb, and tract were not visible, and the pituitary gland was extremely hypoplastic with an ectopic posterior pituitary bright spot. The septum pellucidum was present, but the corpus callosum was very thin (Fig. 1B). Nonetheless, the constellation of optic nerve hypoplasia and hypoplastic pituitary gland was highly suggestive of SOD, so Sanger sequencing of all the coding exons and the exon/intron boundaries of *HESX1* was performed.

### Mutation detection

DNA isolated from the proband was polymerase chain reaction (PCR)‐amplified and sequenced in order to identify potential mutations in the *HESX1* coding exons and flanking introns. Sanger sequencing revealed a heterozygous probably pathogenic variant: c.475C>T (p.R159W) [NCBI Reference Sequence: NM_003865.2] (Fig. 1C). The resulting mutant p.R159W protein was functionally assessed to verify its pathogenicity.

### In vitro mutagenesis

The following PCR primer sets were designed to introduce the c.475 C>T mutation into the wild‐type *HESX1* cDNA via PCR site‐directed mutagenesis: 5‐ATGTCTCCCAGCCTTCAGGA‐3′ and 5′‐CAGTTTTGCACGCCAATTTTGAAACCA‐3′; 5′‐AATTGGCGTGCAAAACTGAAAAG‐3′ and 5′‐TATTCCAGCAGATTTGTGTTG‐3′. The full‐length *HESX1* cDNA containing the mutation was then cloned into the pM vector (Clontech) in frame with the Gal4 DNA‐binding domain (Gal4BD) using EcoRI and SalI restriction enzymes. The product of this expression vector is a fusion protein of the N‐terminal region of Gal4 (1–147 amino acids) and either the WT or mutant *HESX1*. For localization experiments, the full‐length *HESX1* cDNA containing the mutation was cloned into the HA‐pcDNA3.1 vector (Invitrogen) in frame with the HA tag using EcoRI and XhoI restriction enzymes.

### Cell culture and transfection

Human embryonic kidney (HEK)‐293T and CHO (Chinese hamster ovary) cells were maintained in DMEM supplemented with 10% fetal bovine serum, 50 U/mL penicillin, and 50 *μ*g/mL streptomycin. For functional assays, cells were transfected with DNA using Lipofectamine 2000 (Life Technologies) according to the manufacturer's protocol.

### Visualization of subcellular localization

HEK‐293T cells were cultured on gelatine‐coated coverslips and transfected with *HA‐HESX1‐WT* and *HA‐HESX1 p.R159W* expression constructs. 24 h post‐transfection, cells were fixed in 4% PFA and immunofluorescence was performed. Rabbit anti‐HA (Sigma) and goat anti‐rabbit 568 (Alexa Fluor, Thermo Fischer) antibodies were used, respectively, for primary and secondary incubation. Imaging was performed using a Zeiss Axioplan fluorescence microscope.

### Western blots and immunoprecipitation

HEK‐293T cells were transfected with either *HA‐HESX1‐WT* or *HA‐HESX1‐R159W* constructs and harvested 48 h after transfection. Whole‐cell lysates were extracted using basic cell lysis solution (50 mM Tris‐Base pH 7.6, 150 mM NaCl, 1% Triton X‐100) supplemented with a Protease Inhibitor Cocktail (Roche). 10 *μ*g of WT and mutant proteins was separated by SDS‐PAGE (12.5% polyacrylamide), and membranes were incubated with a high‐affinity anti‐HA‐peroxidase rat antibody (Roche) overnight at 4°C. A mouse anti‐glyceraldehyde‐3‐phosphate dehydrogenase (GAPDH) antibody from Millipore was used to assess total loaded protein. Immunoreactive proteins were visualized using the Pierce ECL2 Western Blotting Substrate (Thermo Scientific) according to the manufacturer's instructions. The density of protein bands was quantified using the ImageJ software. For immunoprecipitation, approximately 0.5 mg of total protein from HEK‐293T cells transfected with either *Gal4‐HESX1‐WT* or *Gal4‐HESX1‐R159W* constructs was incubated with anti‐Gal4BD (binding domain) bound to protein G–Sepharose beads at 4°C overnight. Immunoprecipitates were then subjected to Western blot using an anti‐HESX1 antibody. 25 *μ*g (5%) of total protein extract was used as INPUT.

### qRT‐PCR

Total RNA from HEK‐293T cells transfected with either *HESX1‐WT* or *HESX1‐R159W* expression constructs was isolated using the RNeasy micro kit (QIAGEN) with DNaseI treatment. First‐strand cDNA was synthetized with iScript Reverse Transcriptase (BIO‐RAD).

Real‐time PCR was performed with iTaq Universal SYBR Green (BIO‐RAD). Gene expression was determined relative to GAPDH via the ΔCt method. Primers used are here listed. Hesx1‐FW: 5′‐GATCTTCCCAGTGAGACTTC‐3′; Hesx1‐REV: 5′‐CAGGGTAGCAGTTCACTCTA‐3′; GaPDH‐FW: 5′‐ATGACATCAAGAAGGTGGTG‐3′; GaPDH‐REV: 5′‐CATACCAGGAAATGAGCTTG‐3′.

### Luciferase assays

For dual luciferase assay experiments, approximately 50,000 CHO cells per well were seeded into each well of a tissue culture 24‐well plate. Transfection was performed 24 h after seeding, whereas harvesting for analysis occurred 48 h after transfection. To assess the ability of WT and R159W mutant HESX1 proteins to directly bind DNA and repress transcription, a luciferase reporter plasmid containing a SV40 promoter and six copies of the DNA‐binding motif P3 was used, given the high DNA‐binding affinity of HESX1 for P3 11, 16, 20. CHO cells were cotransfected with 80 ng of *Renilla* control vector, 100 ng of the 6P3‐SV40 luciferase reporter, and 100 ng of WT and mutant *HESX1* expression vectors. To evaluate the ability of WT and R159W HESX1 to repress transcription independently from their DNA‐binding activity, we used a luciferase reporter plasmid containing Gal4‐binding sites (Gal4BS‐SV40 luciferase reporter) 11. CHO cells were cotransfected with 80 ng of *Renilla* control vector, 100 ng of Gal4BS‐SV40 luciferase reporter, and 100 ng of *Gal4‐HESX1* expression vectors. In these fusion proteins, the Gal4‐binding domain allows for strong binding, but it is incapable of conferring transcriptional ability unless fused to a protein that can contribute to transcriptional function 21; therefore, WT and R159W HESX1 proteins could be evaluated for their ability to repress transcription independently by their direct binding to DNA. The total amount of transfected DNA was normalized to 350 ng per well by the addition of an empty vector (*pBlueScript*, Stratagene). Cells were harvested and assayed for luciferase activity using the Dual luciferase Reporter Assay System (Promega). Luminescence was measured using a BMG FLUOstar Optima multiplate reader (BMG Labtech Gmbh). Experiments were performed in triplicate and repeated at least three times.

## Results

### Mutation analysis

The R(Arg)159 residue is located in the HESX1 DNA‐binding domain and is highly conserved in vertebrates from fish to humans (Fig. 1D and E). This variant has not been reported in a homozygous state in any of 60,277 individuals in the Exome Aggregation Consortium (ExAC) database 22, which contains sequencing data on individuals without “severe pediatric disease,” but it was found in a heterozygous state in two of 33,194 (~0.06%) non‐Finnish Europeans and 60,277 individuals (1.659 × 10^−5^) in this database. It was predicted by PolyPhen‐2 23 to be “probably damaging” and by SIFT 24 (Sorting Intolerant from Tolerant) to be “deleterious”.

The proband's parents were tested for this specific *HESX1* variant, and his mother was found to carry the same heterozygous variant. The mother herself was born with a birth weight of 2.977 kg, had had one prior normal pregnancy, a minor surgical procedure, and was otherwise healthy. Her height was on the 10th percentile, which was entirely consistent with her expected height based on her parental heights. She had normal optic nerves and underwent a comprehensive evaluation by an endocrinologist that was unremarkable with normal adrenocorticoid, growth hormone, thyrotrope, and gonadotrope axes. She therefore had no clinical nor hormonal evidence of hypopituitarism or optic nerve hypoplasia, but she had never had a brain MRI.

### Functional studies

**Figure 2:**
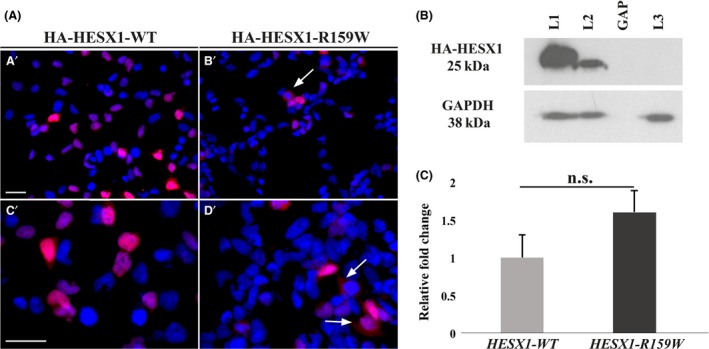
Subcellular localization and Western blot analysis of wild‐type (WT‐HESX1) and HESX1‐R159W proteins. (A) Indirect immunofluorescence on HEK‐293T cells transfected with constructs expressing either HA (human influenza hemagglutinin)‐tagged HESX1‐WT (A′‐C′) or the HESX1‐R159W proteins (C′‐D′). Nuclei are counterstained with DAPI. Both the wild‐type and the mutant proteins localized to the nuclei of transfected cells (red). However, weaker nuclear staining can be observed in cells transfected with the construct expressing HA‐HESX1‐R159W, in combination with infrequent cytoplasmic‐only accumulating cells (B′‐D′, arrows). Scale bar = 50 *μ*m (A′‐B′), 100 *μ*m (C′‐D′). (B) Western blot of total protein extracts from HEK‐293T cells transfected with constructs expressing wild‐type (L1) or mutant (L2) R159W HA‐HESX1 proteins. Note the reduced levels of expression of R159W compared with wild‐type HESX1. L3 represents nontransfected HEK‐293T cells. The expected size of HA‐HESX1 and HA‐HESX1‐R159W proteins is 25 kDa. GAPDH (38 kDa) was used as loading control. (C) qRT‐PCR showing levels of *Hesx1* expression relative to *Gapdh* in HEK‐293T cells transfected with equal amount of *HESX1‐WT* and *HESX1‐R159W* expression vectors. Error bars represent mean ± SEM. The difference between the two samples is not statistically significant.

First, we investigated the cellular localization of the HESX1 p.R159W mutant protein in comparison with HESX1‐WT. Indirect immunofluorescence on transfected HEK‐293T cells revealed weaker nuclear staining in cells transfected with a construct expressing the mutant *HA‐HESX1‐R159W* compared with those transfected with a plasmid expressing the *HA‐HESX1‐WT* protein, in addition to sporadic cytoplasmic‐only accumulation (Fig. 2A, A′‐C′; Fig. 2A, B′‐D′, arrows). This observation was confirmed by Western blot analysis from transfected cells, which showed an approximate 60% reduction in levels of expression of HESX1‐R159W mutant protein compared with HESX1‐WT (Fig. 2B). This reduction was not a transfection artifact because mRNA levels encoding WT and mutant *HESX1* were similar in transfected cells as analyzed by qRT‐PCR (Fig. 2C). These experiments suggest that the R159W substitution leads to the generation of a mutant protein with reduced nuclear localization in transfected cells, suggesting a defect in protein synthesis, degradation, or both.

**Figure 3:**
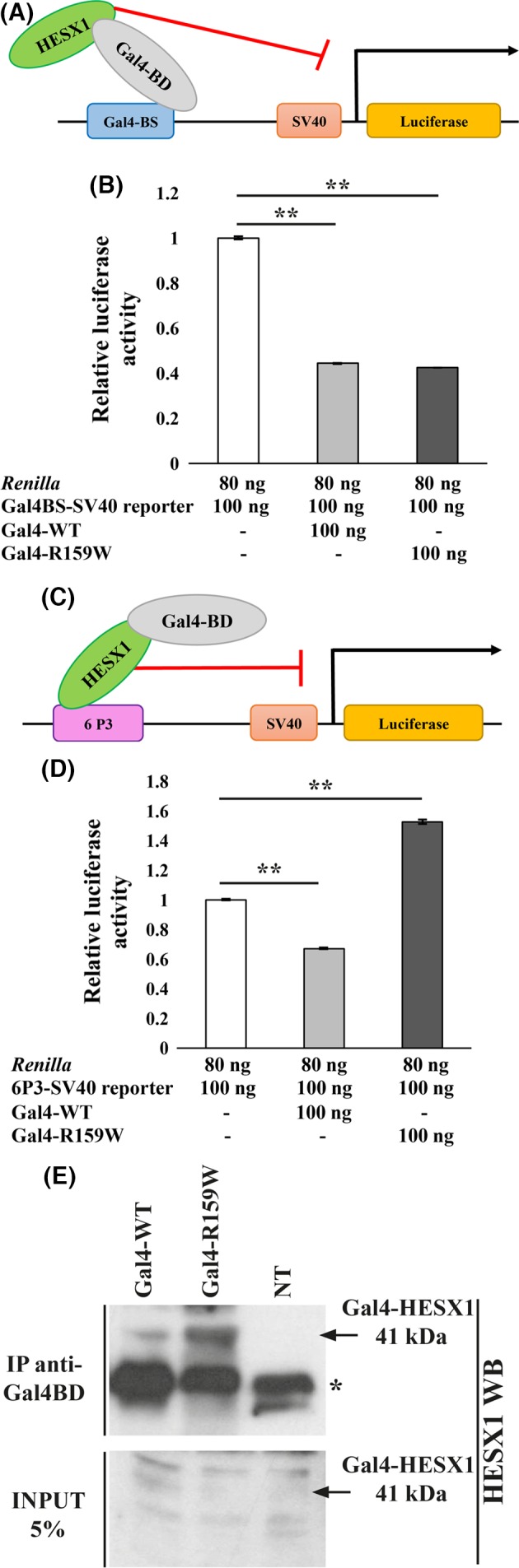
Functional in vitro luciferase assays of wild‐type and R159W‐HESX1 proteins. The luciferase activity of HEK‐293T cells cotransfected with *Renilla* and either the Gal4BS‐SV40 (B) or the 6P3‐SV40 (D) reporter represent the basal level of transcriptional activation in this system and have been used to normalize the subsequent data. Bars represent mean ± 1 SD, *P*< 0.001 (**), *t*‐test. (A) Schematic representation of the mammalian one‐hybrid system using the Gal4‐SV40‐luciferase reporter. In the absence of HESX1, the reporter is constitutively active due to the presence of the SV40 promoter. When the GAL4‐BD (binding domain moiety) fusion protein binds to the Gal4‐binding site upstream the SV40 promoter, HESX1 is able to repress the reporter activity, modulating the luciferase response. In this context, the repressor activity of WT and R159W‐HESX1 can be evaluated independently by their ability to bind DNA. (B) Transfection of constructs expressing either Gal4‐WT and Gal4‐R159W‐HESX1 proteins results in a 55–57% reduction in luciferase activity. (C) Schematic representation of the mammalian one‐hybrid system using the P3‐SV40‐luciferase reporter. In the absence of HESX1 proteins, the luciferase reporter is constitutively active due to the presence of the SV40 promoter. In the presence of the transcriptional repressor HESX1, the direct binding to the P3 consensus site causes a reduction in the basal levels of luciferase transcriptional activity. (D) Transfection with *Gal4‐WT HESX1* expression constructs leads to a 33% reduction in the basal levels of luciferase activity, whereas the mutant construct (R159W) causes a 53% activation. (E) Immunoprecipitation of HESX1 (WT and R159W) proteins fused to the Gal4‐binding domain in HEK‐293T cells. Cells were transfected with plasmids expressing *Gal4‐HESX1‐WT* or *Gal4‐HESX1‐R159W* and immunoprecipitated with an anti‐Gal4‐BD antibody. Immunoprecipitates were blotted and detected with an anti‐HESX1 antibody. Specific immunoreactive bands are indicated with arrows. The 41‐kDa band corresponds to the HESX1 protein (WT or R159W, 22 kDa) fused to the Gal4‐binding domain (19 kDa). NT corresponds to nontransfected HEK‐293T cells. 5% of the total protein extract for each sample was not immunoprecipitated and used as INPUT. Given the low efficiency of the anti‐HESX1 antibody in Western blot alone, together with the low levels of HESX1 protein expression, no Gal4‐HESX1 band (WT or R159W) could be detected in the INPUT. Star(*) marks nonspecific bands, possibly corresponding to the immunoglobulins light chain.

Next, we sought to assess the transcriptional repressing activities of the HESX1‐R159W mutant protein in comparison with HESX1‐WT. CHO cells were transfected with constructs expressing Gal4 fusion proteins (WT and HESX1‐R159W) and a luciferase reporter containing Gal4‐binding sites (Gal4BS‐SV40). In this experimental context, the reporter activity is only dependent on the Gal4 DNA‐binding domain, which when activated allows for stabilization of protein expression, ensuring long half‐lives of the fusion protein's product 25 (Fig. 3A). Indeed, stabilization of the HESX1‐R159W protein by fusion to the Gal4‐binding domain (BD) was demonstrated by immunoprecipitation (IP) with an anti‐Gal4‐BD antibody of protein extracts from HEK‐293T cells transfected with the WT or (p.R159W) mutant *Gal4‐HESX1* constructs, followed by detection of the immunoprecipitated proteins by Western blot using an antibody against HESX1. As shown in Figure 3E, both HESX1 fusion proteins (Gal4‐WT and Gal4‐R159W) could be detected (41‐kDa band corresponding to the fusion product). No band was obtained in the control lane, represented by nontransfected (NT) HEK‐293T cells.

Having shown that protein levels are similar, we assessed the repressing activities of the fusion proteins. Cells transfected with constructs expressing either Gal4‐HESX1‐WT or Gal4‐HESX1‐R159W proteins showed the same levels of repression of reporter activity (0.45 ± 0.004 and 0.43 ± 0.003, respectively) (Fig. 3B). These results suggest that the repressing activities of WT‐HESX1 and HESX1 (p.R159W) mutant proteins are comparable in this Gal4 system.

Next, we decided to evaluate the ability of the HESX1‐R159W mutant protein to bind DNA. We have developed a method to analyze the HESX1 DNA binding activities without the need to perform EMSA, a more hazardous method involving the use of radioisotopes 20. CHO cells were cotransfected with the 6P3‐luciferase reporter, and constructs expressing either Gal4‐HESX1‐WT or Gal4‐HESX1‐R159W proteins. Therefore, in this set of experiments, the reporter activity depends on the direct binding of HESX1 to the P3‐binding sites and is independent from Gal4 (Fig. 3C). Cells expressing Gal4‐HESX1‐WT showed a 33% reduction (0.67 ± 0.006) in the levels of SV40‐mediated transcriptional activation when compared to cells transfected with only the 6P3 reporter vector (i.e., considered as basal level of luciferase activity) (Fig. 3D). In contrast, cells expressing Gal4‐HESX1 (p.R159W) mutant protein failed to repress the basal reporter activation and, indeed, showed higher levels (153%) of luciferase activity (1.53 ± 0.013) (Fig. 3D), possibly due to sequestration of corepressors from the SV40 promoter (see Discussion section). These results suggest that the failure of the HESX1 (p.R159W) mutant protein to repress the transcriptional activation of the P3‐SV40 luciferase reporter is caused by impairment of its DNA binding properties.

## Discussion

In this study, we have identified a novel *HESX1* mutation in a patient with SOD and demonstrated that the resulting mutant protein is functionally compromised.

Firstly, we show that HESX1 (p.R159W) mutant protein localizes to the nucleus, although accumulation of this mutant protein is compromised, as shown by immunostaining and Western blot analyses. It is well known that point mutations in conserved residues can cause protein misfolding, which results in the activation of degradation pathways 26. R159 is a highly conserved residue within the homeodomain from *Drosophila* to humans, and it is present in 347 of 350 homeodomain‐containing proteins 27. It is likely that the R159W substitution leads to compromised protein structure and reduced stability of the mutant protein. However, this is corrected when the mutant protein is fused to the Gal4‐DNA‐binding domain, as this peptide confers greater stability 25.

Secondly, we demonstrate that the R159W substitution does not affect repression because both Gal4‐HESX1‐WT and Gal4‐HESX1‐R159W repressing activities are comparable on a luciferase reporter containing Gal4‐binding sites (Fig. 3B), therefore independently on direct HESX1 or HESX1 (pR159W) binding. This finding is not unexpected, as the *eh1* motif at the N‐terminus of HESX1 p.R159W, which is necessary for interactions with TLE/Grouch corepressors 15, 17, is not affected in the mutant protein.

In contrast, when the repressing activity of the Gal4‐HESX1‐WT or Gal4‐HESX1‐R159W fusion proteins was assessed in a system dependent on direct binding of the HESX1‐WT or HESX1‐R159W to the P3 consensus DNA, the mutant protein showed impaired repression relative to the wild type (Fig. 3D). This suggests that the mutant protein has defective DNA binding properties compared with HESX1‐WT. In fact, the R159W substitution falls within the highly conserved homeodomain, which is essential for the recognition of core‐binding sites (reviewed by Gehring 28). Moreover, SIFT in silico analysis predicts that the R159W substitution is deleterious and could affect protein function 24.

Taken together, our data support previous mouse work showing that point mutations in the HESX1 homeodomain result in loss of DNA binding and protein function 9, 19, suggesting a pathogenic role for the R159W mutation in our patient with SOD. However, the fact that the mother of the affected patient is asymptomatic despite carrying the heterozygous p.R159W suggests that there is variable penetrance of this mutation and other unidentified alleles may also contribute to the observed phenotype. In fact, a multitude of single nucleotide substitutions in different developmental genes have been reported to be associated with SOD 9, 29, 30, 31. Understanding the role of oligogenicity in the generation of SOD and other human phenotypes is a difficult but necessary task for the future. In conclusion, we have identified a novel and pathogenic, most likely by oligogenic interaction, HESX1 (p.R159W) substitution in association with SOD.

Since the submission of this manuscript, the proband's sisters (one born after his passing), aged 3 and 10§ years, have been found to carry the same HESX1 variant. Like their mother, both of them are completely asymptomatic with normal height, normal vision, and normal neurodevelopment, further underscoring the variable penetrance of this variant.

## Authorship

SP and JPMB: designed the experiments. SP: performed the experiments, analyzed the data, and produced the figures. WHT: identified the mutation and provided the clinical data. The three authors wrote the manuscript.

## Conflict of Interest

The authors declare no conflict of interest for the research reported.
